# Eukaryogenesis: The Rise of an Emergent Superorganism

**DOI:** 10.3389/fmicb.2022.858064

**Published:** 2022-05-11

**Authors:** Philip J. L. Bell

**Affiliations:** Microbiogen Pty Ltd., Sydney, NSW, Australia

**Keywords:** evolution, viruses, emergent complexity, eukaryotes, Tree of Life, virocell

## Abstract

Although it is widely taught that all modern life descended *via* modification from a last universal common ancestor (LUCA), this dominant paradigm is yet to provide a generally accepted explanation for the chasm in design between prokaryotic and eukaryotic cells. Counter to this dominant paradigm, the viral eukaryogenesis (VE) hypothesis proposes that the eukaryotes originated as an emergent superorganism and thus did not evolve from LUCA *via* descent with incremental modification. According to the VE hypothesis, the eukaryotic nucleus descends from a viral factory, the mitochondrion descends from an enslaved alpha-proteobacteria and the cytoplasm and plasma membrane descend from an archaeal host. A virus initiated the eukaryogenesis process by colonising an archaeal host to create a virocell that had its metabolism reprogrammed to support the viral factory. Subsequently, viral processes facilitated the entry of a bacterium into the archaeal cytoplasm which was also eventually reprogrammed to support the viral factory. As the viral factory increased control of the consortium, the archaeal genome was lost, the bacterial genome was greatly reduced and the viral factory eventually evolved into the nucleus. It is proposed that the interaction between these three simple components generated a superorganism whose emergent properties allowed the evolution of eukaryotic complexity. If the radical tenets of the VE hypothesis are ultimately accepted, current biological paradigms regarding viruses, cell theory, LUCA and the universal Tree of Life (ToL) should be fundamentally altered or completely abandoned.

## The Dominant Biological Paradigms of Life and Evolution

Three paradigms established in the 19th century, combined with advances in quantitative genetics in the 20th century, led to the dominant ‘textbook’ paradigm of biology where all life is cellular and descends from a common ancestor *via* the neo-Darwinian process of natural selection (e.g., Keeton and Gould, 1986). In the 1830s, the paradigm that all life is composed of cells was established from the work of Jakob Schleiden and Theodor Schwan, and in the 1860s, Pasteur showed that all life descended from previous life, ruling out spontaneous generation. The third paradigm established in the 19th century was Darwin’s theory of natural selection. By providing a mechanism (natural selection) to cause descent with modification, it outlined a scientific basis for understanding the evolution of biological diversity. When Darwin inferred that ‘probably all the organic beings which have ever lived on this earth have descended from some one primordial form, into which life was first breathed’ (Darwin, 1859, p. 484), the paradigms of a universal ancestor and a universal Tree of Life were integrated into Darwinian evolutionary thinking.

In the early 1930s, Fisher published the Genetical Theory of Natural Selection (Fisher, 1930). His infinitesimal model integrated genetics into Darwin’s theory of natural selection and helped resolve the conflict between the Mendelians and the Biometricians. Within Fisher’s model, evolution occurs incrementally as mutations of small effect accumulate in populations and alter phenotypes in response to natural selection. Regarding mutations of large effect, Fisher stated ‘A considerable number of such mutations have now been observed, and these are, I believe, without exception, either definitely pathological (most often lethal) in their effects, or with high probability to be regarded as deleterious in the wild state’ (Fisher, 1930, p. 41). The contributions of Fisher, Wright and Haldane among others led to the Modern Evolutionary Synthesis of the late 1940s and the dominance of this neo-Darwinian paradigm relegated earlier ideas of evolution *via* ‘hopeful monsters’ to the fringes of biological thought (Theissen, 2006).

Initially, genetic drift and neutral evolutionary processes (Kimura, 1968) conflicted with the dominant paradigm but were eventually integrated into evolutionary thinking adding further power to the paradigm. In the 1980s, coalescence theory contributed a framework for examining populations and genes in terms of their evolutionary history (Kingman, 1982; Rosenberg and Nordborg, 2002). The neo-Darwinian Synthesis currently provides a powerful paradigm for understanding how descent with modification can generate biological diversity and today many biologists subscribe to Darwin’s notion of a Last Universal Common Ancestor (LUCA) of all living forms and so subscribe to its corollary, the existence of a universal Tree of Life (Kyrpides et al., 1999; Glansdorff et al., 2008).

Although the Modern Evolutionary Synthesis and the universal Tree of Life are very powerful paradigms, they are under challenge as several major tenets of the synthesis are being questioned (e.g., Doolittle, 1999; Dagan and Martin, 2006; Koonin, 2009; Koonin and Wolf, 2012). One challenge is their incompatibility with endosymbiotic processes operating at the origin of the eukaryotic domain (Koonin, 2009). Since the mitochondrion initially evolved separately from the ancestor of the eukaryotic cytoplasm, it arose *via* a symbiotic event (i.e., saltation) rather than an autogenous incremental process expected under the Modern Evolutionary Synthesis paradigm. An endosymbiotic mitochondrion also makes the eukaryotic cell the product of a merger of at least two separate lineages, which is incompatible with a simple bifurcating tree representing the relationship between eukaryotes and the two prokaryotic domains (Dagan and Martin, 2006). Although a symbiotic origin of chloroplasts was postulated over 100 years ago (Mereschkowsky, 1905), symbiosis ran counter to the dominant evolutionary paradigm of the day and was rejected by the scientific community (Martin et al., 2015). The theory was modernised by Margulis (1975), but in a clear demonstration of how paradigms guide our understanding of phenomena, it took several decades before symbiogenesis became the accepted paradigm for the origin of both chloroplasts and mitochondria (Gray, 1999).

## The “Grand Chasm” Between Eukaryotes and Prokaryotes

The eukaryotic cell is extraordinarily distinct from the much simpler bacterial and archaeal cells of the prokaryotic domains. It possesses not only a nucleus and a mitochondrion, but also a sophisticated endomembrane system, a complex cytoskeleton and a unique sexual cycle, leaving the gap between cells of prokaryotic and eukaryotic design as the greatest chasm in biology. The acceptance of an endosymbiotic origin of eukaryotic mitochondria implies a second distinct kind of cell acquired the mitochondria and became its host during eukaryotic evolution (Martin et al., 2017). Understanding the nature of this host and the origin of its mitochondrion, nucleus and other distinctive features is fundamental to understanding eukaryogenesis. Since archaea and eukaryotes appear to be phylogenetic sister groups (Iwabe et al., 1989) and their information processing machinery is more closely related to each other than either are to the bacteria (Rivera et al., 1998), all plausible eukaryogenesis models must also account for the apparent kinship between the archaeal and the eukaryotic domains. Currently, as anticipated when a paradigm is under challenge, there are many competing models regarding the nature of the original host and how both the nucleus and the mitochondria appeared in the eukaryotic lineage (reviewed in Martin et al., 2015).

Under the historically dominant ‘three-domain’ paradigm, the eukaryotic nuclear line of descent is as old as the archaeal line (Pace, 2009), and the chasm in design has been explained by the hypothesis of a progenote whose cellular design crystallised after the separation of the three domains (Woese, 1998). Despite the nucleus being the defining feature of eukaryotic cells (Stanier and Van Niel, 1962; Sapp, 2005), under the three-domain paradigm, the nuclear membrane is considered by some to be irrelevant in the determination of eukaryotic evolutionary history because the eukaryotic nuclear line of descent has been around since the beginning (Pace, 2009). A notable problem with any model proposing such an early origin of the eukaryotes is the observation that the Last Eukaryotic Common Ancestor (LECA) possessed a mitochondrion, and the mitochondrion is derived from an alpha-proteobacteria (Roger et al., 2017). This means LECA itself cannot be more ancient than alpha-proteobacteria. Geological evidence confirms prokaryotes are far more ancient than eukaryotes since prokaryotes were present by 3.7 billion years ago (Nutman et al., 2016) while the first evidence of eukaryotes occurs only some 1.8 billion years ago (Parfrey et al., 2011). To explain the late appearance of eukaryotes, the *ad-hoc* hypothesis could be made that all non-mitochondrial ‘proto-eukaryotes’ or ‘progenotes’ prior to LECA died out leaving no trace of their existence. However, the alternative must also be considered: the unattested ‘proto-eukaryotes’ or ‘progenotes’ may be purely hypothetical constructs required by the neo-Darwinian paradigm of incremental descent from LUCA and thus may never have existed at all.

Recently, support has been building for an alternative ‘two-domain’ paradigm or Eocyte hypothesis (Rivera and Lake, 1992). In this paradigm, Asgard archaea and all eukaryotes share a common archaeal ancestor (Koonin, 2015; Spang et al., 2015) implying that a bone-fide archaeon related to the Asgard archaea embarked on an evolutionary process that ultimately resulted in the evolution of LECA. In this paradigm, it has been proposed to name the oldest archaeal ancestor whose only living descendants are eukaryotes, the ‘First Eukaryotic Common Ancestor’ (FECA; Eme et al., 2017). Despite speculation about the complexity within the Asgard archaea (Koonin and Yutin, 2014; Spang et al., 2015), culturing of *Prometheoarchaeum syntrophicum* revealed Asgard archaea sit firmly on the prokaryotic side of the grand chasm since they possess a typical small circular prokaryotic genome with no evidence of a nucleus, a mitochondrion or phagocytosis (Imachi et al., 2020). If LECA descended from FECA *via* the small evolutionarily advantageous steps inherent to the neo-Darwinian paradigm, then ‘proto-eukaryotes’ must have existed which were intermediate in design between prokaryotes and eukaryotes. However, there is currently no evidence that an archaeal cell transitioned into a eukaryotic cell *via* any incremental stepwise acquisition of defining eukaryotic features such as the nucleus, endomembrane system, cytoskeleton or mitochondrion. There is only evidence of cells of bona-fide prokaryotic design derived from the archaeal ancestor of FECA and cells of bona-fide eukaryotic design derived from LECA, and nothing to fill the grand chasm in between. As a result, the existence of ‘proto-eukaryotes’ transitional between archaea and eukaryotes remains an unsupported hypothesis within the 2D neo-Darwinian paradigm.

## The Evolution of a Eukaryote From a Bona-Fide Archaeon is a Theoretical Challenge to Incremental Models of Evolution

Explaining how and why the mitochondrion and nucleus appeared in a bona-fide archaeon encounters multiple challenges. For example, the absence of phagocytosis in the Asgard archaea (Imachi et al., 2020) reveals the unresolved challenge of how the mitochondria entered an archaeal ancestor of the eukaryotes (Speijer, 2020). Thus, even though the mitochondrion is clearly an endosymbiont, explaining its origin in bona-fide archaea such as the Asgard group remains an open question. The origin of the nucleus presents even greater challenges. The origin of the nuclear membrane, nuclear pores, an endomembrane system, m7G primed translation, mitosis and the sexual cycle are all unresolved issues associated with the appearance of a nucleus and yet each of these features were present in LECA (Fritz-Laylin et al., 2010; Speijer et al., 2015; Lane, 2017) but are notably absent from prokaryotes such as the Asgard archaea (Imachi et al., 2020).

Being of typical prokaryotic design, the Asgard archaeon *P. syntrophicum* possesses a single circular genome that sits directly in the cytoplasm where translation and general metabolism occur. By contrast, in all eukaryotic cells, a nuclear membrane separates the multiple linear chromosomes from the cytoplasm and ribosomes, introducing a characteristic uncoupling of transcription from translation into all cells of the eukaryotic domain. The nuclear pores are essential to this uncoupling since they enable the export of mRNA to the cytoplasm for translation while simultaneously allowing the import of RNA polymerases from the cytoplasm to enable transcription (Bell, 2020). LECA possessed functional nuclear pores (Neumann et al., 2010) which are complex molecular machines interacting with many other eukaryotic-specific features. A single nuclear pore comprises ∼500 individual proteins through which large molecules such as mRNA and proteins must be actively transported (Kabachinski and Schwartz, 2015). Nuclear pores and the eukaryotic clathrin vesicular trafficking system also appear to share a common evolutionary origin and were both part of an early membrane-curving module and internal membrane system in eukaryotes (Devos et al., 2004). An unanswered puzzle is how and why would a nuclear pore evolve to export mRNA or import RNA polymerase across a membrane in an archaeon before a nuclear membrane existed? This is particularly challenging since archaea possess a perfectly adequate system of coupled transcription and translation where mRNA already interacts directly with the translational apparatus (Benelli and Londei, 2011). Alternatively, how could a nuclear membrane evolve in an archaeon before nuclear pores were functional? Without nuclear pores, mRNA could not be exported from the nucleus and no RNA polymerase could be imported to the nucleus and thus, the nucleus could not function.

In bona-fide archaea such as the Asgard group, translation relies on direct recognition of mRNA by the ribosomal apparatus (Benelli and Londei, 2011). By contrast, in eukaryotes, the m7G cap of mRNA primes eukaryotic mRNA for splicing, export from the nucleus and translation in the cytoplasm (Bell, 2020). Although many archaeal and eukaryotic translation factors such as eIF1A, eIF2, eIF2B, eIF4A, eIF5B and eIF6 share a common ancestry to the exclusion of the bacteria, translation of m7G capped mRNA in eukaryotes requires an extra set of eukaryotic-specific initiation factors. These include eIF5, eIF4G, eIF4B, eIF4H, eIF3 and eIF4E, of which eIF4E is particularly important since it directly recognises the m7G cap and allows initiation of translation in the cytoplasm (Jagus et al., 2012). An unexplained puzzle is why would an m7G cap evolve in an archaeon before the nucleus appeared, and what would be the selective advantage of an m7G cap if it evolved before there was eIF4E to recognise the cap? Conversely, why would eIF4E evolve before the m7G cap evolved to prime the splicing and export of mRNA from the nucleus?

Typically, prokaryotes such as Asgard archaea replicate by binary fission which is very distinct from eukaryotic mitosis since their circular genomes are not segregated by a dynamic cytoskeleton. In eukaryotic mitosis, the linear eukaryotic chromosomes are condensed, and the tubulin-based spindle apparatus of the dynamic cytoskeleton binds to the centromeres and segregates the condensed chromosomes to the polls of the cell prior to cell division. How and why an archaeon genome would switch from binary fission to the complex process of mitosis where separate linear chromosomes are condensed around a nucleosome scaffold, align at the equator of the cell during metaphase and are segregated by a dynamic cytoskeleton using centromeres and a spindle apparatus is unresolved and a challenge to explain under an incremental model of evolution.

Finally, a notorious ‘queen of evolutionary problems’ related to the origin of the nucleus is the unresolved paradox of the origin of the eukaryotic sexual cycle (Bell, 1982). For the sexual cycle to function, two highly complex integrated but temporally and mechanistically unrelated processes must occur. Firstly, meiosis must occur to convert a diploid cell into four haploid daughter cells. This complex process is achieved by a single cycle of chromosomal replication generating a nucleus with 4 N ploidy and is followed by two mitosis-like cell divisions reducing the ploidy of the four daughter cells to 1 N. Like mitosis, it requires nucleosome-based condensation of chromosomes, centromeres for segregation and a dynamic cytoskeleton, but unlike mitosis which simply replicates the genome, meiosis is a part of a conceptually very different process that ‘jumbles up’ the genome and reduces the ploidy of the resulting cells by half. To complete the sexual cycle and restore diploidy, mating between two haploid cells of opposite mating types must occur which requires both gamete recognition and a fusogen to fuse the haploid cells together to generate a diploid cell. The origin of this process is a paradox that has defied any generally accepted explanation for over 50 years and in part revolves around the challenge of determining which came first, meiosis that allows haploid gametes to be formed from a diploid or syngamy that allows 1 N haploid gametes to mate and create a 2 N diploid in the first place.

According to the modern evolutionary synthesis, incremental changes leading to the complex, unique and interrelated eukaryotic systems associated with the nucleus must have each provided an immediate selective advantage to an archaeal cell. Arguing these innovations were beneficial because they allowed the *future* evolution of complexity in the eukaryotic domain is clearly a teleological argument. It is particularly thought-provoking to explain these discontinuities in terms of incremental benefit when it appears that the eukaryotic system evolved only once in over 3.7 billion years and left no currently recognised intermediates, while the prokaryotic system remained highly efficient and conserved by the bacterial and archaeal domains for over 3.7 billion years.

## Where do the Undead Viruses Fit Into the Standard Evolutionary Paradigm?

Under the current textbook paradigm of biology, the fundamental unit of all life is the cell and all cellular life descends from LUCA *via* incremental change. Under this paradigm, the metaphor of a single universal Tree of Life is one of the most important organising principles of biology representing the relationships between all life forms. Where do the viruses fit under this paradigm? Viruses are not cellular and fail to meet this definition of life. Since viruses also lack ribosomes, they cannot be placed on the universal Tree of Life which was originally defined based on phylogenetic analysis of the ribosomal apparatus (Woese and Fox, 1977; Moreira and López-García, 2009). However, viruses cannot simply be dismissed as non-living material since both viruses and cells store genetic information in nucleic acids, are composed of genes, encode proteins and replicate and are subject to Darwinian evolutionary forces. If all life is dogmatically defined as cellular, the position of viruses in biology becomes highly ambiguous. As a result, even by 1927, there was an extensive history of debates on whether viruses are alive (Rivers, 1927) and the question is still debated today (Claverie and Abergel, 2016; Forterre, 2016).

The current ambiguity of the viral paradigm has deep roots in the history of biology. In Latin, virus means something like ‘venom’ and a virus was originally a disease producing fluid (Lwoff, 1957). Upon the discovery of cellular pathogens (e.g., fungi), cell-based organisms were ‘deprived of their ancestral right to be called viruses’ (Lwoff, 1957) and the term ‘filterable virus’ was applied to all non-cellular infectious agents that passed through a Chamberland filter (Rivers, 1927). Under this definition, viruses were infectious agents too small to be seen using 19th century microscopes and according to the ‘cell theory paradigm’ they were not cellular and therefore not alive. Although the term ‘filterable virus’ was abbreviated to ‘virus’, ‘filterable’ remained part of the viral paradigm for decades. It took the discovery of the giant Mimivirus (La Scola et al., 2003) to emphasise how flawed this ‘filterable’ assumption was. Since discovering viruses could be as complex as cells, scientists have found that giant viruses are abundant in many ecosystems (Al-Shayeb et al., 2020; Moniruzzaman et al., 2020; Boratto et al., 2022). In a demonstration of the power of a paradigm to influence the way that discoveries are interpreted, the largest known viruses, the Pandoraviruses, were not recognised as viruses when first discovered but rather were described as ectosymbionts (Scheid et al., 2008). In 2013, these ectosymbionts were recognised as giant viruses and found to possess a genome of 2.5 megabase pairs, which is even larger than some parasitic eukaryotes (Philippe et al., 2013).

Alive or not, viruses are evolving biological entities that appear to share a long evolutionary history with cellular organisms (Durzyńska and Goździcka-Józefiak, 2015) and may have even emerged prior to the origin of the DNA-based cellular domains (Nasir and Caetano-Anollés, 2015). It has also been proposed that the emergence of genetic parasites like viruses is inevitable due to the instability of parasite-free states (Koonin et al., 2017) and that a prokaryotic genome free of genetic parasites will degenerate due to Muller’s ratchet (Iranzo et al., 2016). Thus, viruses and cells are likely to be interdependent entities that emerged in concert with each other, and their evolution has been intertwined ever since (Durzyńska and Goździcka-Józefiak, 2015; Krupovic et al., 2019).

Viruses are also extremely abundant and of major evolutionary significance. In the oceans, viruses are the most abundant ‘lifeforms’ with an estimated 4 × 10^30^ viruses killing an estimated 20–40% of marine bacteria daily (Suttle, 2005, 2007). In aquatic environments, it has been discovered that they out-number cellular organisms by between 10- and 100-fold and play a very significant role in ecosystems limiting the abundance of any one species and thus maintaining ecological diversity (Fuhrman, 1999). The role of viruses in prokaryotic evolution is exemplified by their role in transduction of genes including those for antibiotic resistance as well as phage encoded toxins (Díaz-Muñoz and Koskella, 2014). In eukaryotes, much of the genome is composed of viral elements. For example, the human genome is composed of > 20% LINES and ~ 13% SINES, both of which are repetitive elements derived from retroviruses (Liehr, 2021). In animals, the ARC genes underlying synaptic plasticity required for memory are clearly derived from retroviruses (Budnik and Thomson, 2020) and the mammalian placenta relies on endogenous retroviral elements to function (Blond et al., 2000). Significantly, HAP2 which enabled mating to occur in LECA (Wong and Johnson, 2010) is structurally a close match to a wide range of viral fusogen proteins supporting a possible viral origin of this fundamental eukaryotic feature (Valansi et al., 2017). It has also been argued that viruses were essential to the origin of life as we know it since they may have been involved in the invention of DNA, enabling the transition from the RNA world to the DNA world (Forterre, 2002).

Finally, the ‘all life is cellular’ paradigm leads to strange linguistic anomalies that reflect the failure of this paradigm to adequately describe the diversity of life on earth. A comparison between *Rickettsia bellii* and the giant Mimivirus illustrates the problem. The Mimivirus was discovered infecting *Acanthamoeba polyphaga* (La Scola et al., 2003), a host in which *R. bellii* can also replicate (Ogata et al., 2006). Thus, both organisms have evolved to replicate in the same host, are membrane bound obligate internal parasites, have similar sized complex DNA genomes, transcribe DNA into RNA and encode enzyme complexes including RNA polymerases, DNA polymerases and topoisomerases. Yet *R. bellii* is alive and the *Mimivirus* is, for want of a suitable English word, ‘undead’. Thus, under the current paradigm, we can say a virus is not alive, but we cannot say what it is. Further examples of these anomalies include a viral life cycle for something that is not alive and viral vaccines that can be ‘live or dead’. By defining all life as cellular, the viruses become some kind of undead zombies that can be killed even though they are not alive!

## The Viral Eukaryogenesis Model for the Emergence of a Eukaryotic Superorganism

The appearance of stromatolites by 3,700 million years ago (Nutman et al., 2016) shows that prokaryotic life rapidly emerged on earth. However, 3,700 million years later, stromatolites remain some of the most complex structures built by prokaryotes, indicating the limited ability of prokaryotes to evolve the organismal complexity that characterises the eukaryotic domain. Similarly, despite being related to FECA, modern Asgard archaea retain a simple prokaryotic level of organisation (Imachi et al., 2020) and have shown little indication of any special evolutionary potential for evolving eukaryotic level complexity over the last couple of billion years. In contrast to the prokaryotes, the first eukaryotes emerged only some 1.8 billion years ago (Parfrey et al., 2011), and yet have evolved from a ‘protozoan-like’ LECA into complex multicellular organisms including self-aware humans. Furthermore, since all eukaryotes descend from LECA that was already eukaryotic (Lane, 2017), we only have evidence that this occurred once in the 3.7-billion-year history of life on earth. An outstanding question for evolutionary biology to answer is how and why did a single lineage apparently evolve beyond a prokaryotic level of complexity?

According to the Viral Eukaryogenesis hypothesis (Bell, 2001), the answer to this question is that the eukaryotic cell is an emergent superorganism (Bell, 2020), and its emergent nature allowed the evolution of complexity. Emergent phenomena occur when simple components interact to produce a level of complexity greater than the sum of its parts. For example, snowflakes possess intricate 6-fold symmetry and yet all snowflakes are composed of simple water molecules. The complex snowflake design is thus an emergent property of the way water molecules interact. By analogy, the eukaryotic cell is derived from three simpler components whose evolutionary potential is an emergent property of the way they interact. Thus, it was not the intrinsic properties of the Asgard archaea that provided any special ability to evolve complexity, it was the way that an Asgard archaeon was colonised by a virus and interacted with a bacterial endosymbiont that led to an emergent superorganism which had greater evolutionary potential than any of its parts.

**Figure 1:**
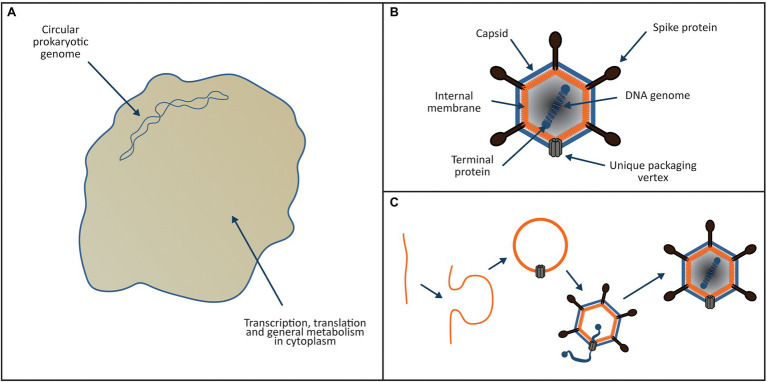
Proposed ancestors of the eukaryotic cytoplasm and nucleus. **(A)** Archaeal ancestor of the eukaryotic cell was a bona-fide archaeon of the Asgard group. It possessed a small circular genome located directly in the cytoplasm, transcription and translation were linked and it encoded its own translational apparatus, ESCRT system and like *P. syntrophicum* (Imachi et al., 2020); it most likely was a syntroph. **(B)** The viral ancestor of the eukaryotic nucleus (and NCLDV viruses) was a PRD1-like tectilivirus that infected the ancient Asgard ancestor of the eukaryotes. It possessed a small linear chromosome (~15 kb) with inverted terminal repeats, formed icosahedral capsids and incorporated a membrane derived from the host plasma membrane into its capsid. **(C)** As per the model proposed by Hong et al. (2014), the tectilivirus had the ability to sharply curve membranes to create clathrin-like pits generating membrane bound vesicles that formed the basis of the viral capsids.

**Figure 2:**
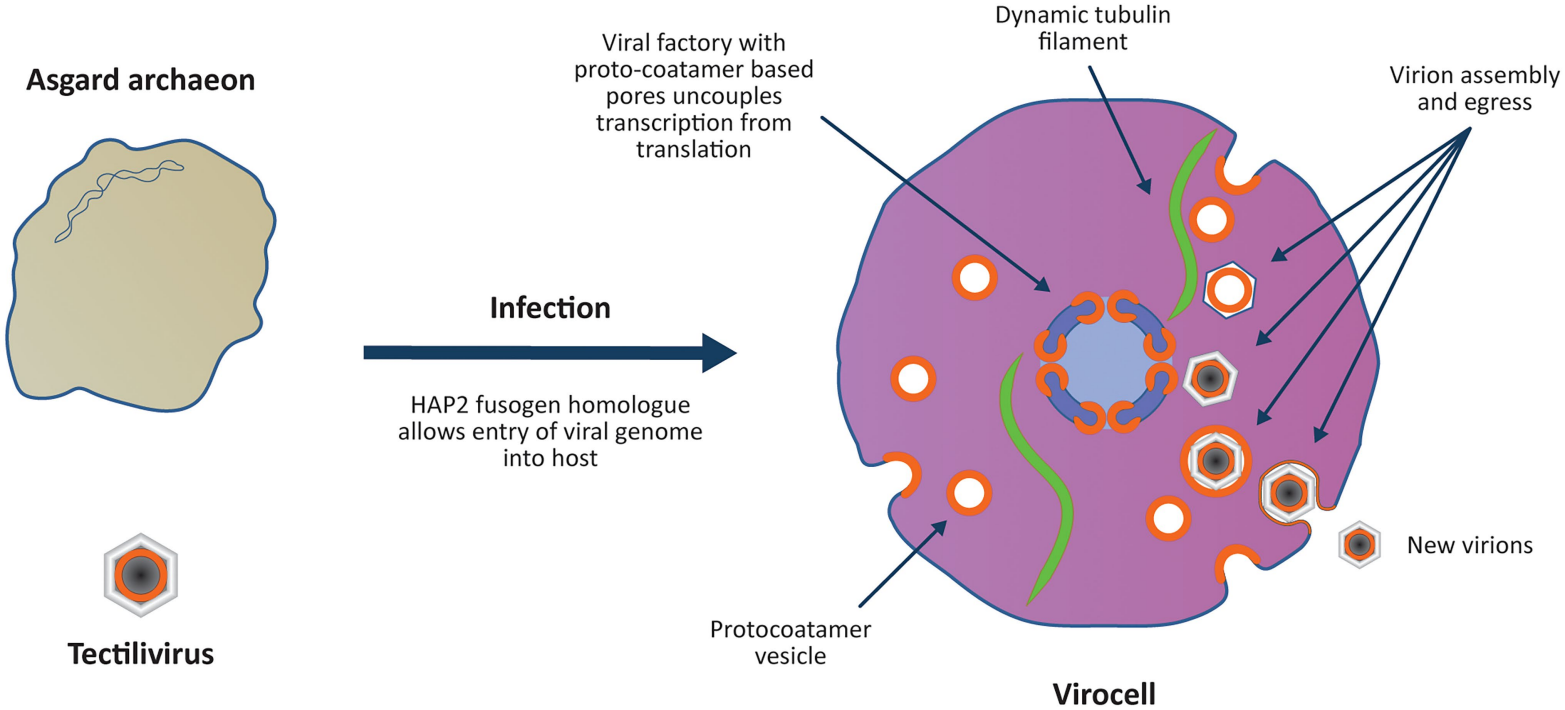
Viral colonisation of the Archaeal host introduces multiple “eukaryotic” features in a single saltational event. The virus genome enters the host archaeon by fusing its internal membrane with the host membrane using an ancestral HAP2 fusogen homologue. As observed with *Sulfolobus* infections, the virocell may have become significantly enlarged upon viral infection due to modification of the host’s cell cycle and ESCRT system (Liu J. et al., 2021). The virus establishes a viral factory protecting the viral genome from host defence mechanisms and introduces an uncoupling of transcription from translation. Viral ‘protocoatamers’ sharply bend membranes producing clathrin-like pits that break off to form ‘coated vesicles’ for constructing new virions (*cf.* Hong et al., 2014). The protocoatamers are also used to construct pores in the viral factory allowing controlled transport of proteins and RNA in and out of the viral factory. Tubulin encoded by the virus forms a dynamic cytoskeleton that positions the viral factory in the centre of the cell (Chaikeeratisak et al., 2017). The coated vesicles are treadmilled along the tubulin cytoskeleton towards the viral factory where the virions are assembled (Chaikeeratisak et al., 2019) and DNA is condensed on nucleosomes and packaged into the virion. Protocoatamer constructed vesicles allow egress of virions through the plasma membrane without disrupting the plasma membrane.

**Figure 3:**
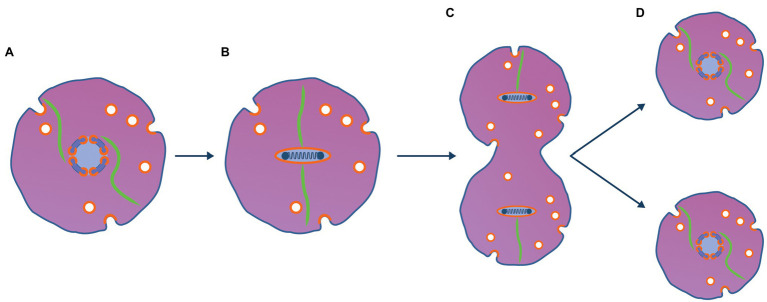
Viral method to partition viral chromosome to daughter cells is ancestral to mitosis. **(A)** A copy number control mechanism based on centromeres and partitioning proteins analogous to those seen with phage and plasmids maintains the viral genome as a single copy [(1 N); Ebersbach and Gerdes, 2005]. **(B)** Prior to the archaeal host entering a cell division, the viral genome is replicated (becomes 2 N) and is condensed using the viral nucleosomes used to package the genome into virions. When the 2 N genome is condensed, viral transcription and translation cease and the tubulin cytoskeleton binds to the viral genome at the centromere. **(C)** Prior to binary fission using typical archaeal mechanisms, the virus encoded dynamic tubulin proteins bind to centromeric sequences of the viral genome pulling the genome to opposite poles of the cell. **(D)** After the cell has divided the viruses re-establish 1 N viral factories and resume transcription and translation.

**Figure 4:**
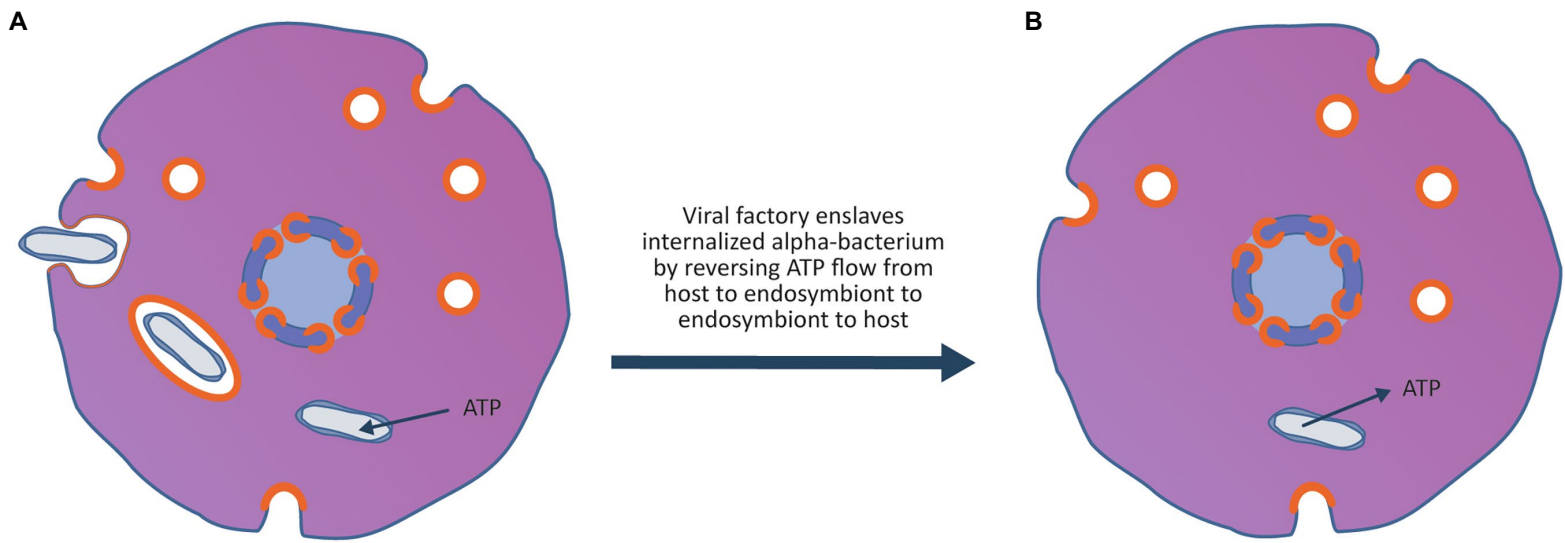
Enslavement of a parasitic alpha-proteobacterium by the virocell leads to the evolution of an emergent superorganism. **(A)** An alpha-proteobacteria evolves the ability to enter the cell exploiting the vesicle system of the archaeal virocell. Once inside the cell, the bacterium can escape the vesicle and enter the host cytoplasm. After entry to the cytoplasm, the bacterium imports ATP and nutrients from the host cytoplasm and becomes an internal energy parasite of the virocell. These internal bacterial parasites are ancestors of the *Rickettsia*-like eukaryotic parasites as well as the mitochondria. **(B)** The rare event that made the process of eukaryogenesis unique in the history of life on earth occurred when the viral factory evolved the ability to enslave the mitochondrion, forcing it to stop importing ATP and nutrients from the host cytoplasm and instead export ATP and nutrients into the host cytoplasm. By taking control of the metabolism of the bacteria, the bacterium was changed from an energy parasite into the endosymbiotic powerhouse of the superorganism. The advantage to the viral factory of enslaving the bacterium ensured that the virocell and the enslaved bacterium embarked on an evolutionary trajectory of increasing metabolic and genetic integration, leading to the evolution of an emergent superorganism.

**Figure 5:**
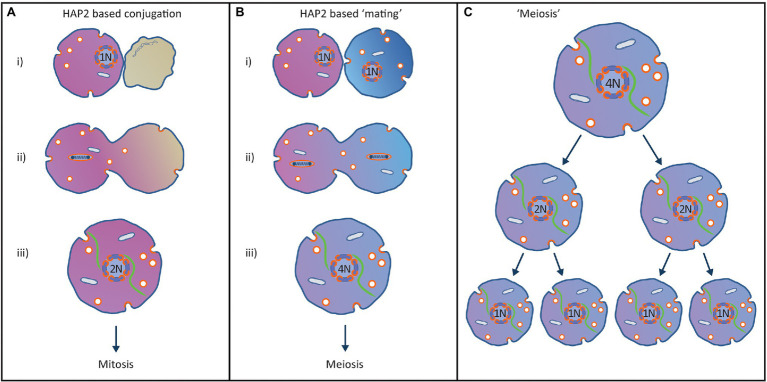
Proposed origin of the eukaryotic sexual cycle from a HAP2-based conjugation process to co-transfer virus and mitochondrion to new hosts. **(A)** Horizontal transmission virus/endosymbiont to new hosts. (i) Superorganism encounters suitable host cell and recognizes that it is not infected with the virus. (ii) Prior to conjugation the viral genome replicates and condenses in preparation for fusion with the new host. The plasma membranes of the two cells subsequently fuse using the HAP2 fusion protein used by the virus in its standard infection cycle. (iii) After complete fusion of the cells, the viral factory is re-assembled, and the viral factory is centred in the cell using the tubulin cytoskeleton. The 2 N viral genome can enter a single mitosis-like cell division controlled by the viral copy number control system that maintains the viral genome at 1 N. **(B)** Origin of the eukaryotic sexual cycle. (i) Two divergent but closely related superorganisms recognise each other as a potential host but fail to recognise the other host is already infected by a closely related virus. This results in both superorganisms initiating the conjugation process. (ii) Prior to conjugation, the viral genomes replicate and condense in preparation for transfer to new hosts and the plasma membranes of the two cells fuse using the HAP2 fusion protein encoded by the virus. (iii) After complete fusion of the cells, the viral factories are re-assembled, and the viral factories are centred in the cell using the tubulin cytoskeleton. The two 2 N viral factories fuse to create a single 4 N viral factory containing two homologous pairs of viral chromosomes. **(C)** Due to the copy number control mechanisms evolved by the virus to maintain a low copy number, the 4 N viral genome must enter two mitosis-like cell divisions without viral genome replication to reduce the viral genome back to 1 N, resulting in completion of a primitive eukaryotic sexual cycle.

**Figure 6:**
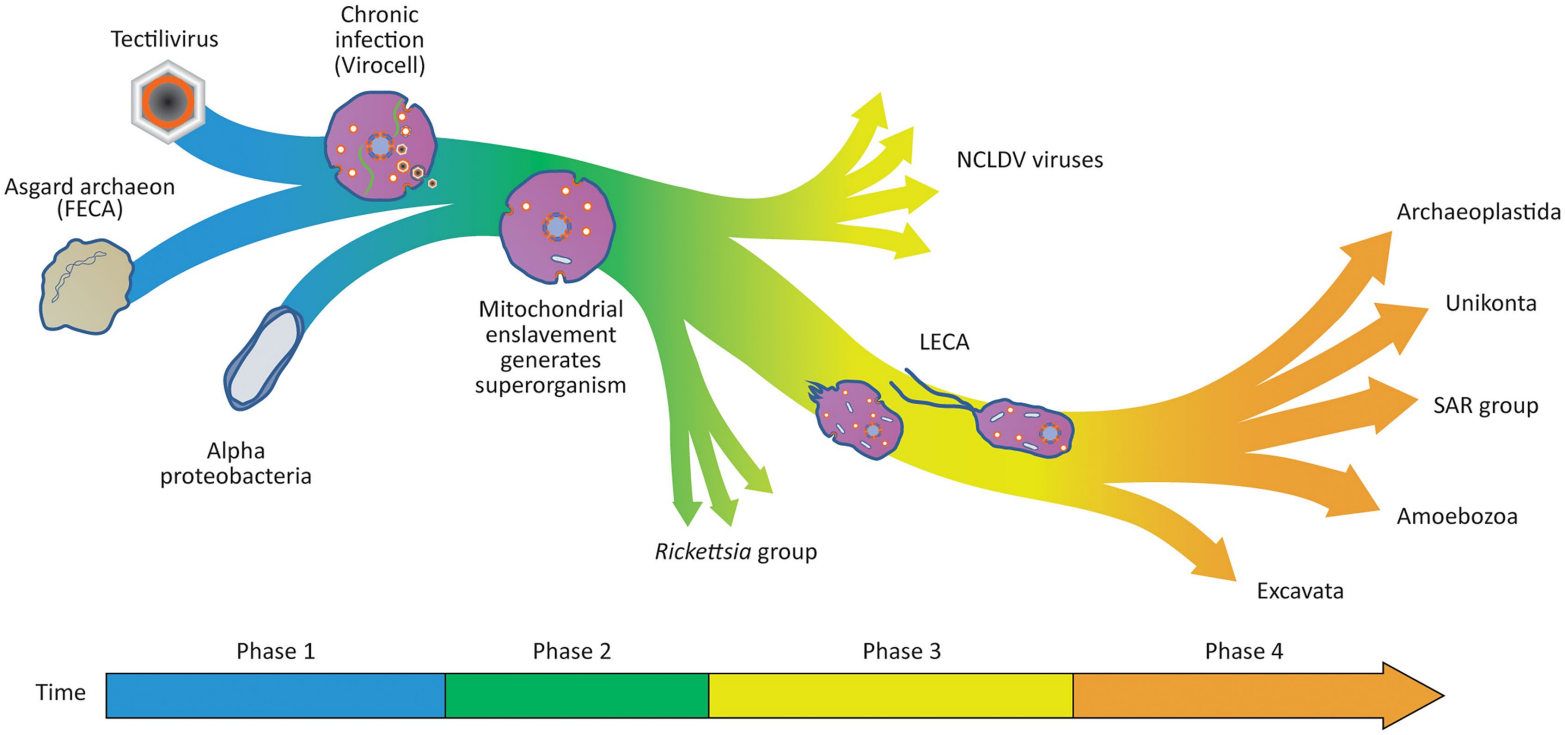
Viral Eukaryogenesis model for the origin of the eukaryotic cell. In Phase 1 of the eukaryogenesis process, the chronic infection of an Asgard host results in the generation of a virocell. Within the virocell, the viral factory introduces a separation of transcription from translation and several other eukaryotic features that contribute to its future potential to evolve complexity including an endomembrane system and a dynamic cytoskeleton. In Phase 2 of the eukaryogenesis process, an alpha-proteobacteria evolves to infect the virocell through the vesicle system that allows the Tectilivirus to chronically infect the archaeal host. The rare and defining step of eukaryogenesis occurs when the viral factory enslaves one of the bacterial parasites converting the virocell into a three-component superorganism with emergent properties. The enslaved endosymbiont produces energy and raw materials to sustain the viral factory in the host cell, making it advantageous for the virus and endosymbiont to be located in the same cell. In Phase 3 of the eukaryogenesis process, there is a continuing transfer of genes from the enslaved endosymbiont to the viral genome as it takes over complete control of the endosymbiont’s metabolism. A similar process results in the transfer of a complete translation apparatus and basic metabolism from the host archaeon to the viral genome. During this process, the superorganism evolves the ability to differentiate into an amoeboid predator when prey is abundant, and a flagellated motile form to allow dispersion and infection of new hosts. By the time LECA appears, only 69 genes remain in the endosymbiont genome, and the archaeal host genome is completely lost, resulting in a protozoan-like eukaryotic predator. In Phase 4, phagocytosis allowed the superorganism to become an amoeba-like predator that also possessed a unique mode of evolution introduced by the sexual cycle. This led to an evolutionary arms race selecting for larger and more complex eukaryotic organisms resulting in the evolution of the diverse range of eukaryotes observed today. Unlike the origin of LECA as a superorganism, the evolution of the eukaryotes after the appearance of LECA is well described by the neo-Darwinian synthesis, and thus, a tree metaphor is a useful description of the relationships between all members of the eukaryotic domain.

According to the Viral Eukaryogenesis model presented in Figures 1–6, FECA belonged to the Asgard group and was a bona-fide archaeal ancestor of the eukaryotic cytoplasm and translational apparatus. Prior to the eukaryogenesis process (Figure 1A), FECA was of typical prokaryotic cellular organisation. It possessed a small circular genome, encoded its own translational apparatus and ESCRT system and was most likely a syntroph like *P. syntrophicum* (Imachi et al., 2020). The common ancestor of both the NCLDV viruses and the eukaryotic nucleus was a member of the Tectiliviricetes (Figure 1B), chosen partly because the Tectiliviricetes and the NCLDV group share a common ancestor (Woo et al., 2021). By analogy with the phage PRD1 (a modern Tectiliviricetes), the viral ancestor of the nucleus possessed a linear chromosome (~15 kb) with inverted terminal repeats, formed icosahedral capsids and incorporated a membrane derived from the host plasma membrane into its capsid. As per the model for the PRD1 life cycle proposed by Hong et al. (2014), the virus could sharply curve the host membranes to create clathrin-like pits that budded inwards from the cell membrane forming vesicles (Figure 1C).

Eukaryogenesis was initiated by the colonisation of an Asgard host by the Tectilivirus virus as a low copy number lysogen. It is proposed that the viral DNA entered the host cytoplasm by fusing the viral and host membranes using an ancestor of the eukaryotic HAP2 fusogen. The virus lysogenised the host, and by setting up a permanent viral factory in its cytoplasm, it converted the archaeon into a virocell (Figure 2). As observed with *Sulfolobus* infections, the virocell may have become significantly enlarged upon viral infection due to modification of the host’s cell cycle and ESCRT system (Liu J. et al., 2021). By analogy to phage phiKZ viral factories, the viral factory prevented attack by the host immune system (Mendoza et al., 2020) and separated viral transcription from translation (Chaikeeratisak et al., 2017). The virus encoded a dynamic tubulin cytoskeleton which both positioned the viral factory in the middle of the cell and treadmilled viral capsids to the factory (Chaikeeratisak et al., 2019). The m7G capping system was a viral invention that targeted viral mRNA for export into the cytoplasm where the host ribosomes had been reprogrammed to selectively translate m7G capped mRNA. A viral ancestry of mRNA capping is supported both by its absence from any known prokaryotes, and phylogenetic evidence showing that *Mimiviridae* and the eukaryotic nucleus obtained m7G capping from a common ancestral source that predated the origin of LECA (Bell, 2020). Phylogenetic evidence of a viral ancestry of the eukaryotic alpha-DNA polymerase has also been inferred, providing evidence others have used to also propose a viral ancestry of the eukaryotic nucleus (Takemura, 2001). The virus sharply bent the host membrane to produce clathrin-like pits (*cf.* Hong et al., 2014) which interacted with the host ESCRT system to produce lipid vesicles that were incorporated into virions. The viral membrane bending protein was the ancestral ‘protocoatomer’ proposed by Devos et al. (2004) and was used for both vesicle formation and the construction of pores in the viral factory. Since modern NCLDV viruses of the *Marseilleviridae* group form eukaryote-like nucleosomes that are packaged with viral DNA into virions (Liu Y. et al., 2021), it is proposed that eukaryotic nucleosomes evolved as a viral innovation to package viral DNA into virions. Phylogenetic analysis is consistent with this proposal since *Marseilleviridae* histone doublets and eukaryotic histone doublets both derive from an ancestral source that predated the origin of LECA (Erives, 2017). Secondary infection of the virocell by the same virus was prevented by superinfection immunity mechanisms analogous to those observed in modern phage, which lysogenise their hosts as autonomous low copy number plasmids. For example, phage N15 which lysogenises its host as a low copy number linear plasmid contains a primary immunity locus (*immB*) which prevents superinfection by other N15 phage (Ravin et al., 1999).

As shown in Figure 3, mitosis is proposed to have emerged from the viral method to partition its linear chromosome to daughter cells and was analogous to the way viral lysogens such as phage N15 (Ravin, 2011) and phage P1 (Łobocka et al., 2004) are maintained indefinitely as low copy number plasmid-like replicons (Ebersbach and Gerdes, 2005). Several genes encoded and expressed by prophages in their lysogenic state ensure that prophage genomes such as P1 can be maintained at about one copy (1 N) per host bacterial chromosome (Łobocka et al., 2004). These genes include copy number control systems as well as active partitioning systems to ensure the physical segregation of plasmids to daughter cells (Bouet and Funnell, 2019). During the cell’s growth phase, the 1 N viral factory is centred in the cell by the viral tubulin cytoskeleton. When the virocell initiated the archaeal binary fission process, the viral genome responded by replicating to generate a 2 N viral factory. Subsequently, it would be condensed and inactivated using viral packaging nucleosomes and the tubulin cytoskeleton would bind to the viral centromere and partition the two replicated genomes to opposite poles of the cell. After the host cell completed binary fission, the viral genome would decondense and the 1 N viral factories would be re-established. In support of this model, when *Clostridium botulinum* phage c-st lysogenises its host as a low copy number lysogen, it uses a protein homologous to tubulin to partition its plasmid-like genome to daughter cells (Oliva et al., 2012).

As shown in Figure 4, after permanent colonisation of the archaeal host by the viral factory, an alpha-proteobacterium ancestral to the *Rickettsia* group evolved the ability to exploit the viral vesicle system to become an internal parasite of the Asgard virocell. As proposed by Wang and Wu (2014), the ancestor of the mitochondria was an energy parasite that could import ATP and other nutrients directly from the host cytoplasm. The event that made eukaryogenesis unique in history occurred when the viral factory enslaved an internal bacterial parasite. By changing the bacterium from a consumer of the host’s ATP and nutrients to a producer of ATP and nutrients for the host, it created a three-component superorganism consisting of the archaeal host, the viral lysogen and the enslaved bacterium. The enslavement of the mitochondrial ancestor firmly linked its fate with that of the virus and provided selective pressure for the evolution of a conjugation-like process to co-transfer the virus and enslaved endosymbiont to new hosts.

As shown in Figure 5, meiosis and the sexual cycle originated from a conjugation process that allowed co-transfer of the virus and the enslaved mitochondria to new hosts. When a superorganism encountered an uninfected host cell, a conjugation process would start in order to transfer both the virus and enslaved mitochondrion to a new host (Figure 5A). The viral genome would replicate, condense onto nucleosomes as per the mitotic cycle and the infected cell would use the HAP2 fusogen to fuse the two cells together. After fusion, a 2 N viral factory would be re-assembled, which would then enter a mitosis-like cell division. The two daughter cells produced would each contain a 1 N viral factory and an enslaved mitochondrion, allowing the viral factory to continue its advantageous relationship with the enslaved mitochondrion.

The eukaryotic sexual cycle arose when two closely related superorganisms failed to recognise that both were infected with homologous viruses (Figure 5B). Since the superinfection immunity function failed to recognise the other host was infected with a similar virus, both hosts entered the conjugation process. Consequently, both viral genomes would replicate then condense and the plasma membranes of the two superorganisms would fuse using the HAP2 fusogen. After fusion, both viral factories would be re-assembled, and both 2 N viral factories would be centred in the fused cell using the tubulin cytoskeleton. When the two 2 N viral factories contacted each other at the centre of the cell they would fuse to create a single 4 N viral factory containing two pairs of homologous but not identical viral genomes. The copy number control system used in mitosis (Figure 3) would prevent the viral genomes replicating until the 4 N superorganism had gone through two mitosis-like cell divisions and produced four daughter cells with 1 N viral factories (Figure 5C). This conjugation system provides a basis for the eukaryotic sexual cycle including mating of cells, and a meiotic cycle using the same segregation system evolved in mitosis (Bell, 2006, 2013). This aspect of the model is supported by the observation that viral fusogens and the HAP2 fusogen used by LECA share a common origin (Valansi et al., 2017).

The overall process is summarised in Figure 6 and starts with a virus colonising an archaeon to create a virocell and ends with the emergence of LECA, a superorganism whose evolutionary potential exceeds those of its parts. In a first phase of eukaryogenesis, colonisation of the archaeon by the virus created a virocell simultaneously introducing multiple unique eukaryotic features in a single saltational event. These features include: linear chromosomes, the separation of transcription from translation, the m7G capping system, a dynamic cytoskeleton, mitosis, nucleosomes and a primitive protocoatamer system. In a second phase of the eukaryogenesis process, the vesicle system of the colonised archaeon allowed the evolution of specialised parasitic alpha-proteobacteria capable of entering the virocell’s cytoplasm. One of these parasites was eventually enslaved by the nuclear ancestor creating a superorganism consisting of three separate organisms of three very different origins. Alpha-proteobacteria that were not enslaved by the viral factory remained parasites of the superorganism and their descendants are the *Rickettsia*-like bacteria that infect modern eukaryotes. Viral relatives of the nuclear ancestor also maintained the ability to infect new superorganisms and their descendants became the modern NCLDV viruses. The third phase of eukaryogenesis was characterised by the transfer of genes from both the mitochondrion and the host archaeon to the viral factory as each organism became increasingly integrated into a single superorganism. During this phase, the mitochondrion was reduced to an organelle encoding only 69 genes (Roger et al., 2017) with the rest of the ~1,000 genes of its proteome transferred to the viral/nuclear genome. The viral factory was simultaneously acquiring genes from the host archaeal genome, ultimately including both a complete translational system and a basic set of metabolic pathways. This process allowed the remnants of the archaeal genome to become redundant and ultimately lost as a separate replicon. This aspect of the model is supported by the observation that many modern NCLDV genomes encode genes involved in central carbon metabolism, including most of the enzymes for glycolysis, gluconeogenesis, the TCA cycle and the glyoxylate shunt (Moniruzzaman et al., 2020). Modern NCLDV viruses also have a track record of acquiring genes associated with translation. For example, the Tupanvirus has assembled multiple genes involved in translation including 70 tRNA, 20 tRNA synthetases, and 11 factors for all translation steps and factors related to tRNA/mRNA maturation and ribosome protein modification (Abrahão et al., 2018). Finally, during this third phase and before the emergence of LECA, the superorganism differentiated into two specialised forms, an amoeboid form capable of bacterial predation and a motile form to disperse into new environments and infect new cells when conditions became marginal. During the fourth phase of eukaryogenesis, phagocytosis allowed the superorganism to become an amoeba-like predator possessing a unique mode of evolution introduced by the sexual cycle, and this led to an evolutionary arms race selecting for larger and more complex eukaryotic predators resulting in the modern eukaryotic divisions observed today. In the model, LECA was a free living protozoan similar to modern members of the Excavata such as *Naegleria gruberi* which are capable of differentiating into motile flagellated forms or amoeboid forms (Fritz-Laylin et al., 2010).

## The Origin of the Eukaryotic Cell as an Emergent Superorganism Requires Radical Changes to the Dominant Biological Paradigm

If the basic tenets of the Viral Eukaryogenesis hypothesis are ultimately accepted, paradigms regarding a universal Tree of Life, LUCA, viruses and cell theory should be fundamentally altered or completely abandoned. That is, while the Viral Eukaryogenesis hypothesis provides a plausible and testable model for the evolution of the eukaryotes, it is built on an evolutionary paradigm that is a radical departure from current paradigms of biological evolution. Under the Viral Eukaryogenesis hypothesis, the origin of the eukaryotes becomes more reminiscent of Goldschmidt’s ‘hopeful monsters’ (Theissen, 2006) than the neo-Darwinian model of incremental descent from LUCA.

The concept of a universal Tree of Life assumes descent from a universal ancestor. This long-cherished paradigm is not valid if the eukaryotes are an emergent superorganism since eukaryotes will not be specific relatives of either the archaeal domain or the bacterial domain but are an amalgam of three separate organisms including a virus. While phylogenetic analysis of genes derived from the archaeal host will place the eukaryotic superorganism within the archaeal domain, this simply represents the fact that one of the components of the superorganism was an archaeon. Similarly, analysis of genes derived from the bacterial endosymbiont will place the eukaryotes within the bacterial domain, but again this simply represents that fact that a second component of the superorganism was a bacterium. Phylogenetic analysis of genes of viral origin will be particularly problematic under the current paradigm since viruses cannot be placed on the Tree of Life and are not even defined as living organisms. Thus, while there are still three distinct cellular domains under the Viral Eukaryogenesis paradigm, they did not descend from a single common ancestor *via* incremental descent and the metaphor of a single bifurcating tree representing their evolutionary history is invalid.

**Figure 7:**
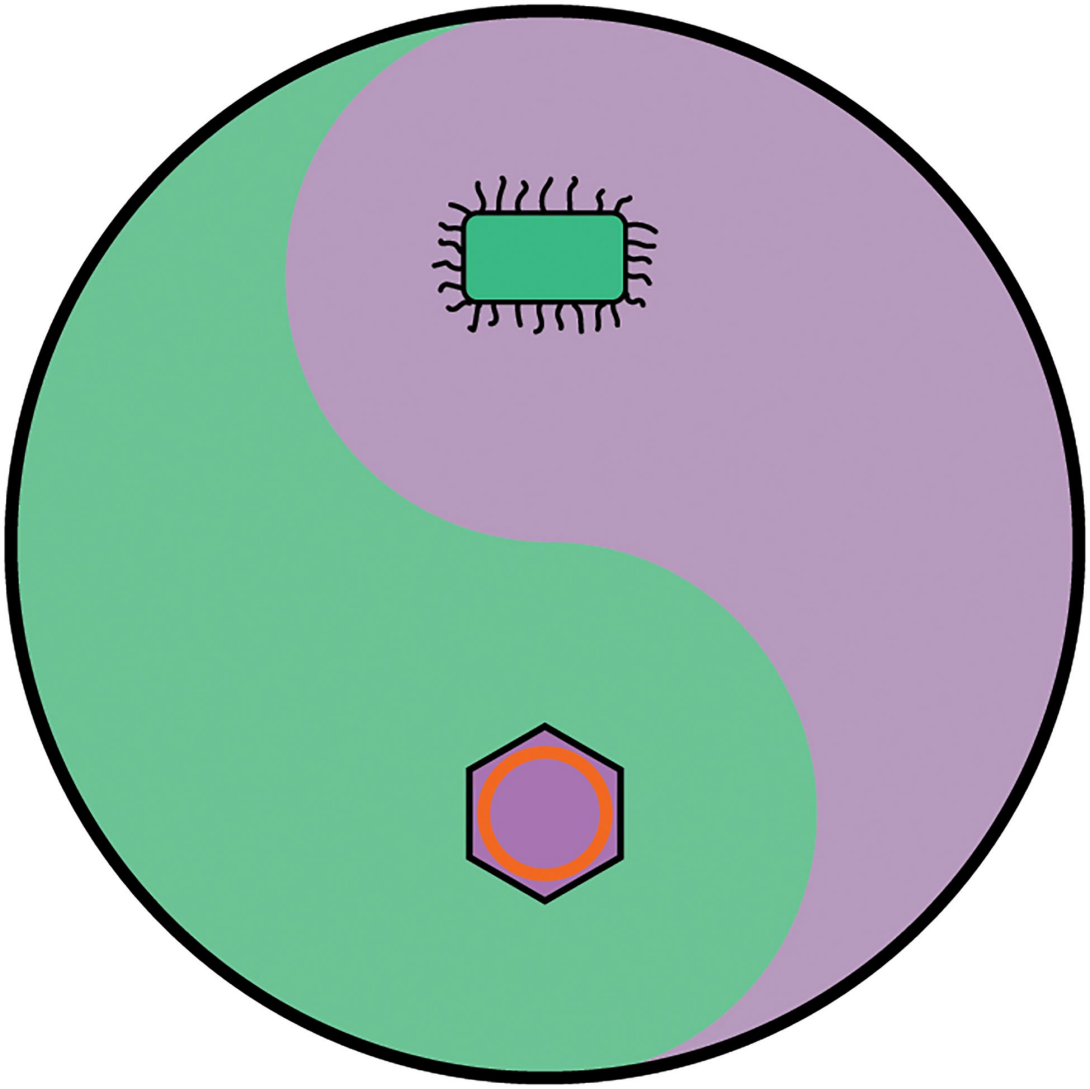
The yin-yang metaphor for life. Organisms are defined as belonging either to the cellular domains or the viral hordes. The cellular domains include all members of the Bacterial, Archaeal and Eukaryotic domains, and are characterised by possessing DNA genomes which encode all the genetic information to construct functional ribosomes. The ability to synthesise proteins gives them a high degree of autonomy and allows them to encode all the enzymes and proteins to maintain and replicate a cell. The Viral hordes are composed of viruses, plasmids, transposons and retrotransposons and are characterised by being mobile genetic elements that are dependent of being in a cellular environment to display the features associated with life. The organisation and modes of evolution of the cellular domains and the viral hordes are of opposite nature, and yet are interdependent. Since viruses and cells may not be able to evolve without each other, it is anticipated that there have always been both these interdependent life forms since life began and may even have been characteristic of life before it evolved into recognisably modern forms.

Viruses currently fail to meet the definition of life and yet they cannot simply be dismissed as non-living material. If cells rely on viruses to avoid Mullers ratchet (Iranzo et al., 2016) and have been intertwined with cells since the beginning (Durzyńska and Goździcka-Józefiak, 2015), viruses and cells can be considered two contrasting yet interdependent life forms. To resolve the debate about whether viruses are alive, it is proposed that the dogmatic 19th century definition that ‘life is cellular’ (based of the resolving power of an optical microscope) is replaced with the equally dogmatic definition that ‘life is either cellular or viral’. Under this definition, it is proposed that the universal Tree of Life as a metaphor for biology is replaced with the metaphor of a yin-yang pair (Figure 7). A yin-yang pair is chosen because a yin-yang pair encompasses the idea that viruses and cells have opposite but interdependent natures. Furthermore, it does not require viruses and cells to descend from a common ancestor, and nor does it propose that viruses are a missing link between life and non-life. Like a previous proposal (Raoult and Forterre, 2008), the cellular life forms are those based on cells that contain ribosomes (the three domains), whereas viral life forms (the viral hordes?) are mobile genetic replicators such as the viruses, plasmids, phage, transposons, retrotransposons and viroids that co-evolve with cells and are a major source of evolutionary innovation. In a significant paradigm shift for biology, this proposal would unify all life (both viruses and cells) into a single unit, composed of two very distinct groups of organisms that are not related to each other in a tree-like fashion but are related to each other by their opposing but interdependent nature. In this new paradigm, ‘viruses are life, but not as we know it’.

Finally, it should be noted that Darwin applied his theory of natural selection to members of the eukaryotic domain, almost exclusively plants and animals. Unlike the contested relationship between viruses, bacteria, archaea and eukaryotes, there is mounting evidence that all modern eukaryotes descend from a single Last Eukaryotic Common Ancestor (Lane, 2017), and thus, all eukaryotes can and should be placed on a single eukaryotic Tree of Life. Since Darwin was essentially ignorant of bacteria (Davies, 2009), his assumption that all the organisms with which he was familiar descended from a common ancestor *via* the process of natural selection has essentially been vindicated, and as he intuited over 150 years ago, their evolutionary relationships are best described in a tree-like fashion.

## Data Availability Statement

The original contributions presented in the study are included in the article/supplementary material, further inquiries can be directed to the corresponding author.

## Author Contributions

PB conceived and wrote the paper.

## Conflict of Interest

PB was employed by the company Microbiogen Pty Ltd.

## Publisher’s Note

All claims expressed in this article are solely those of the authors and do not necessarily represent those of their affiliated organizations, or those of the publisher, the editors and the reviewers. Any product that may be evaluated in this article, or claim that may be made by its manufacturer, is not guaranteed or endorsed by the publisher.
